# Water-Absorbing Bioadhesive Poly(Acrylic Acid)/Polyvinylpyrrolidone Complex Sponge for Hemostatic Agents

**DOI:** 10.3390/bioengineering9120755

**Published:** 2022-12-02

**Authors:** Tomoko Ito, Shingo Yamaguchi, Daisuke Soga, Keisuke Ueda, Takayuki Yoshimoto, Yoshiyuki Koyama

**Affiliations:** 1 Obara Hospital Research Institute, 3-28-16, Honcho, Nakano-ku, Tokyo 164-0012, Japan; 2 Department of Immunoregulation, Institute of Medical Science, Tokyo Medical University, 6-1-1 Shinjuku, Shinjuku-ku, Tokyo 160-8402, Japan; 3 Japan Anti-Tuberculosis Association, Shin-Yamanote Hospital, 3-6-1, Suwa-cho, Higashimurayama, Tokyo 189-0021, Japan

**Keywords:** hydrogels, bioadhesion, hemostasis, Poly(acrylic acid), polyvinylpyrrolidone

## Abstract

Background: Poly(acrylic acid) (PAA) is a water-soluble synthetic polymer with tissue-adhesive properties. When PAA is mixed with polyvinylpyrrolidone (PVP) in water, it forms a water-insoluble precipitate that neither swells nor adheres to tissues. Methods and Results: We developed a novel solid/solution interface complexation method to obtain a water-swellable PAA/PVP complex. First, PAA solution was dried up in a vessel to form a film. The PAA film was then immersed in an aqueous PVP solution to obtain a highly swollen PAA/PVP hydrogel. Heat drying of the hydrogel yielded a transparent film, while freeze-drying the hydrogel provided a soft sponge. Both the PAA/PVP film and sponge could be re-swelled by water to obtain a bioadhesive gel. A relatively larger specific surface area of the sponge than that of the film led to a more rapid swelling and water absorption behavior and quick adhesion to tissues. The addition of hyaluronic acid (HA) improved the mechanical characteristics of the sponges. PAA/PVP/HA sponges had low cytotoxicity, and they exhibited high hemostatic efficiency in clinical studies after dialysis treatment or tooth extraction, even in patients on antithrombotic drugs. Conclusions: Such bioadhesive materials consisting of low-toxicity polymers have a high potential for use in medical hemostatic devices.

## 1. Introduction

Biocompatible hydrogels that adhere to living tissues have high potential for application in medical devices, such as hemostatic agents, wound dressings, or controlled drug-release systems [1,2,3,4,5,6]. If bioadhesive hydrogels can be enzymatically or hydrolytically degraded inside the body into safe water-soluble materials, they can be implanted or left inside the body as a localized hemostasis or bio-adhesion barrier used in surgery without requiring removal [7,8,9,10]. Poly(acrylic acid) (PAA) is a water-soluble synthetic polymer that has been known to adhere to the mucous membrane [11,12,13,14,15,16], skin [17], bone [18], and teeth [19]; therefore, it can be a potential candidate for a self-adhesive hemostatic or wound-dressing agent [20,21].

However, the high water-solubility of PAA limits its use in solid medical devices. Water-insoluble crosslinked PAAs were prepared by the copolymerization of acrylic acid monomers with bifunctional monomers, such as 4,4′-di(methacryloylamino)azobenzene, divinylbenzene, or 2,5-dimethyl-1,5-hexadiene [22,23]. Crosslinked PAAs were also obtained by γ-irradiation of aqueous PAA solutions [24]. These three-dimensionally crosslinked PAA gels absorb water to form swollen hydrogels depending on the crosslinking density. However, in some cases, the crosslinking agents used to prepare the hydrogels may cause some toxicity. Additionally, chemically crosslinked polymers are generally non-biodegradable, which may become a problem when implanted in the body [25].

In contrast, PAA physically binds to polyvinylpyrrolidone (PVP) to form a water-insoluble complex when mixed in water [26,27,28]. Such physically crosslinked gel can be obtained without requirement of crosslinker, and thus the risk of toxicity induced by the agent should be avoided [29].

One of the main interactions between PAA and PVP is hydrogen bonding between the carboxyl groups in the side chains of PAA and pyrrolidonyl pendant groups in PVP. Thus, when the PAA/PVP complex gel is placed under physiological conditions, the carboxyl groups of PAA are gradually neutralized by the permeating cations, causing dissociation of the complex into the original water-soluble polymers [30,31,32]. Thus, the interpolymer complex of PAA and PVP is considered to be a hydrolytically degradable medical device. When aqueous solutions of PAA and PVP are mixed, a polymer complex immediately forms and is precipitated. A water-insoluble transparent film is obtained by drying up the complex precipitates. However, the PAA/PVP complex obtained by simple mixing of the polymer solutions is neither soluble nor swollen in water. In addition, the PAA/PVP complex does not exhibit adhesive properties to biotissues. The bio-adhesive ability of PAA is attributed to the presence of free carboxylic acid groups. However, in the PAA/PVP complex, the main chains of PAA and PVP are aligned parallel to each other and bound through hydrogen bonding, and the carboxyl groups of the PAA molecules are mostly consumed by hydrogen bonding with the pyrrolidonyl groups of PVP. However, restriction to the movement of the polymer segments during complex formation may prevent the highly ordered parallel alignment of the main chains, leaving the carboxyl groups free in part. The unbound carboxyls impart not only bio-adhesivity, but also water-swelling properties owing to their high hydrophilicity.

To hinder the parallel alignment of the polymer main chains, we developed a solid–solution interface complex formation procedure. First, PAA solution was dried up to form a film, and then PVP solution was added to the PAA film. Briefly, an aqueous or ethanol solution of PAA was placed in a plastic cup. Drying the solution resulted in a PAA film attached to the bottom. An aqueous PVP solution was then added to the PAA films. PAA gradually swelled into the solution, simultaneously forming a complex with PVP, leading to the formation of a soft swollen hydrogel. Drying up the hydrogel yielded a transparent PAA/PVP complex film, which again absorbed water to form a highly swollen hydrogel. The re-swollen gel exhibited high adhesion properties to bio-tissues.

The bioadhesive PAA/PVP film demonstrated favorable hemostatic effects in animal experiments. When the swellable PAA/PVP complex film was placed on a bleeding site, it soon swelled to form a hydrogel, stuck to a hemorrhaging spot, and effectively arrested the bleeding [32,33]. Considering the clinical applications of the complex to control the bleeding site, quick swelling is required before heavy bleeding occurs. Heavy or prolonged bleeding is undesirable, and excessive blood causes a decrease in the adhesion strength of the PAA/PVP complex (Figure S1). The PAA/PVP complex should absorb water and form a hydrogel that exhibits bioadhesion properties. Thus, a complex that absorbs water and immediately forms a swollen gel is desirable for achieving quick hemostasis. For rapid control of bleeding, the PAA/PVP complex in a porous sponge would be favored because a sponge material has a relatively large specific surface area for water absorption and requires a relatively shorter contact time to swell up than a film. A flexible sponge is also preferable for managing a large curvature surface, such as a fingertip, or for inserting it into the narrow tooth extraction socket. In this study, we prepared spongy PAA/PVP complexes by freeze-drying the swollen complex hydrogel under various conditions, and their application as hemostatic devices for dialysis treatment or tooth extraction was examined in clinical studies.

## 2. Materials and Methods

### 2.1. Materials

PAA for pharmaceutical use, Carbopol 934P NF, was purchased from Kobayashi Perfumery Co., Ltd. (Tokyo, Japan). PVP, Kollidon 90 (MW = 360,000 g/mol) was provided by BASF Japan Ltd. (Tokyo, Japan). Sodium hyaluronate (HA) fermented by Streptococcus zooepidemicus HA-LQH (MW = 1,200,000–2,200,000 g/mol) was purchased from Kewpie Corporation (Tokyo, Japan). A synthetic leather, Protein LeatherTM PBZ13001-BK (5 mm thick), was obtained from IDEATEX JAPAN Co., Ltd., Tokyo, Japan.

### 2.2. Fluorescence Labelling of PAA

PAA (105.4 mg) was dissolved in 8 mL DMSO and mixed with Texas Red™ cadaverine (Texas Red™ C5) (TR) (1 mg). 1-Ethyl-3-(3-(dimethylamino)propyl)carbodiimide (186 mg) [34] in 3 mL of DMSO was subsequently added to the mixture. After standing at 25 °C overnight in the dark, it was dialyzed against water for 4 days. The precipitated polymer was collected via centrifugation and washed with 30% NaCl. After washing with NaCl solution ten times, the polymer became highly swellable in water. It was swollen in water and dialyzed against running water for two days, and then against deionized water for three days. Finally, the TR-labeled PAA was freeze-dried to a red-purple sponge, with a yield of 56.1 mg.

### 2.3. Preparation of Water-Swellable PAA/PVP Complex

Typically, an ethanol solution of PAA (1.5 wt.%, 1.2 mL) was added to a 25 mm × 25 mm plastic tray and allowed to dry at 70 °C to form a transparent film attached to the bottom of the tray. Then, the aqueous PVP solution (1.98 wt.%, 1.4 mL) was added at a PAA:PVP ratio of 1:1 (by moles of repeating units; 1:1.54 by weight). After standing at 25–27 °C for a given period, the swollen PAA/PVP complex was cooled to −60 °C and freeze-dried using a freeze-dryer CS-55 (SAKUMA Seisakusho Co. Ltd., Tokyo, Japan). The PAA/PVP complex in film form was prepared by air-drying the swollen PAA/PVP complex at room temperature instead of freeze-drying. The complexes containing HA were prepared via pre-addition of HA to the PVP solution before addition to the PAA film. To prepare a stick-shaped complex sponge (7 mm × 7 mm × 25 mm), a silicone mold with a thickness of 7 mm and through-holes of 25 mm length and 7 mm width attached to the polypropylene sheet was used instead of the plastic tray.

### 2.4. Measurement of the Mechanical Strength of the PAA/PVP Sponge

Mechanical strength of the PAA/PVP sponge was evaluated using a compression test carried out at a temperature of 25 °C and relative humidity of 30%. The PAA/PVP complex sponge (7 mm × 7 mm × 25 mm) was set in a tensile testing machine (ZTA-50N digital force gauge and MX2-500N test stand (Imada Co., Ltd., Aichi, Japan)) with a vertical long axis, and its compressive strength was measured at a pressing rate of 50 mm/min.

### 2.5. Measurement of the Adhesion Strength of the PAA/PVP Sponge or Film

#### 2.5.1. Shear Adhesion Strength

The adhesion strengths of the complex sponges were measured at 25 °C using a tensile testing machine (ZTA-50N digital force gauge and MX2-500N test stand). Synthetic leather was used as an adhesion substrate. Leather was wiped with ethanol before use to improve the wettability of the surface. A film (12 mm × 10 mm × 0.12 mm) or sponge sheet (12 mm × 10 mm × 1.9 mm) of the PAA/PVP complex was attached to the upper support of the apparatus using double-sided tape. A square piece of synthetic leather (10 mm × 10 mm) was fixed to the vertical side of the 2 kg aluminum block with double-sided tape and placed right under the complex. The upper clamp was lowered to adjust the height of the sample to match that of the leather piece. Water was applied evenly to the leather pieces by holding the complex sample away from the leather. The sponge was then brought into contact with the leather piece for a given period, and the upper support was raised at 50 mm/min. The force applied to detach the complex from the leather or break the complex was measured.

#### 2.5.2. Pull-off Adhesion Strength

A stick-shaped PAA/PVP complex sponge (7 mm × 7 mm × 25 mm) was cut in the middle of the long axis. A cut piece was fixed by a clamp to the upper support, with the cross-section facing downwards. A synthetic leather piece (10 mm × 10 mm) was attached to the top surface of the aluminum block. A given amount of water was applied to the leather pieces, and the upper support was lowered at 30 mm/min until the pressure force reached 2 N. The support was stopped, and the pressing force was maintained at 2 N for 10 s. It was then raised at 15 mm/min, and the force applied to detach or break the complex was recorded.

### 2.6. Evaluation of the Cytotoxicity of PAA/PVP Complexes

Cytotoxicity of the PAA/PVP/HA complex sponge was examined by placing the solid sponge directly onto the cultured cells as follows. Mouse fibroblasts were generated from BALB/c mice, as previously described [32]. Cells were seeded onto 24-well plates at 3 × 10^4^ cells per well and cultured for 4 days in Dulbecco’s modified Eagle’s medium (DMEM) supplemented with 10% fetal bovine serum (FBS), penicillin G sodium (100 unit/mL), and streptomycin sulfate (0.1 mg/mL). After removal of the primary growth medium, a PAA/PVP/sponge (2 mg) was softly placed on the cells. Fresh DMEM (1 mL) with FBS and antibiotics was gently added to the wells. After 3 days incubation at 37 °C with 5% CO2, the culture medium was removed, and cell viability was measured using the WST-1 assay.

### 2.7. Hemostatic Effect of PAA/PVP Complexes on Mice

All animal experiments were conducted in accordance with the protocol approved by the Tokyo Medical University Animal Committee (No. R3-0122). Female BALB/c mice were purchased from SLC (Shizuoka, Japan). A vein of the femur of mice (15 weeks old) was exposed under general anesthesia and incised. A PAA/PVP complex sponge stick containing HA (7 mm × 7 mm × 2.5 mm) was placed on the hemorrhage part, and a hemostatic effect was observed for 20 min.

### 2.8. Clinical Study of the Hemostatic Effect of PAA/PVP Complexes

The clinical studies were approved by the Ethics Committee of Shin-Yamanote Hospital (Protocol No:12004), and informed consent was obtained from all subjects. Pharmaceutical grade sodium hyaluronate (from chicken comb) produced by Shiseido Company Limited (Tokyo, Japan), was used in the clinical research. One side of the PAA/PVP/HA sponge sheet (25 mm × 25 mm) not touching the wound was backed with a cotton lining and applied for hemostasis after blood dialysis. After dialysis was completed, the puncture site was disinfected with an iodine solution. A sponge sheet was attached to the wetted skin surface around the injection area. After removal of the needle, the sheet was softly pressed for 60 s. It was then gently peeled off, and hemostasis was examined.

Hemostasis was performed after tooth extraction in 11 patients. Two of them were prescribed warfarin, and international normalized ratios (INRs) were 1.69 and 1.72, respectively. Soon after the extraction, the PAA/PVP/HA complex sponge sheet (2.5 mm × 2 mm) was placed on the bleeding socket, and the hemostatic effect and recovery conditions were observed. Treatment after tooth extraction was also performed with PAA/PVP/HA in stick form (7 mm × 7 mm × 25 mm) in two patients.

## 3. Results and Discussion

**Figure 1:**
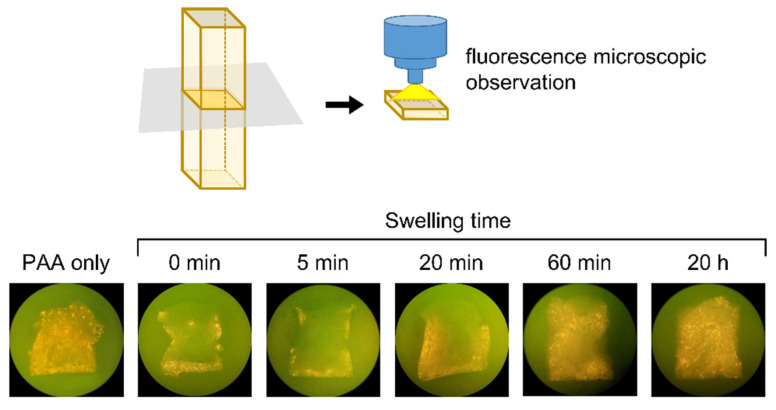
Fluorescence microscopic images of cross-section of the PAA/PVP complexes.

When an aqueous PVP solution was added to a dried solid PAA film attached to the bottom of the plastic tray or inner side of the silicone mold, the PAA film started to swell immediately to produce a highly swollen PAA/PVP hydrogel. While air-drying the hydrogel resulted in a transparent film, a white flexible sponge was obtained by freeze-drying the swollen complex gel. The swelling behavior in the stick-shaped silicone mold was observed using fluorescence-labeled PAA. After addition of the aqueous PVP solution to the labeled PAA film formed inside the silicone mold, the mixture was left to stand at 25 °C for a given period. The swollen gel was rapidly frozen at −60 °C and freeze-dried. The resulting sponge was cut in the middle of the long axis, and the cross-section was observed using a fluorescence microscope (Axiovert 40 CFL, Carl Zeiss Co., Ltd., Wetzlar, Germany). Fluorescence was observed at the edge of the cut surface of the sponges, which were immediately frozen after the addition of PVP. Fluorescently labeled PAA then gradually spread over the cross-section and covered almost the entire surface within 60 min (Figure 1).

**Figure 2:**
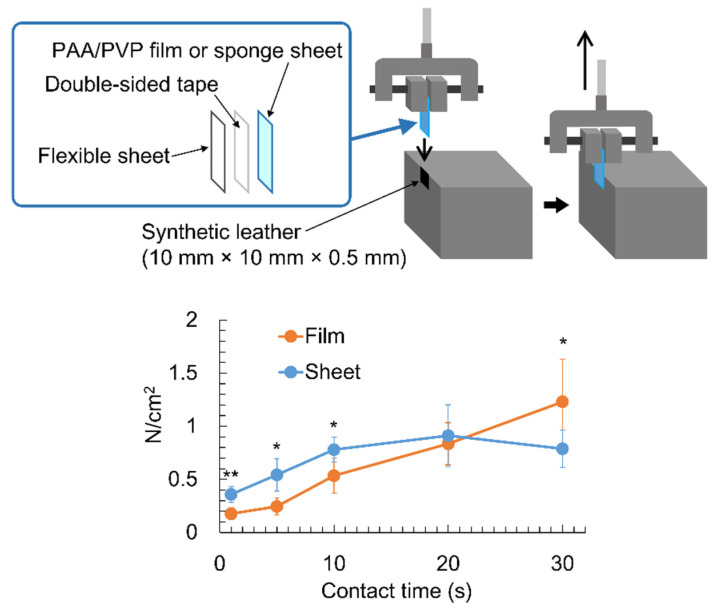
Measurement of shear adhesion strength of the PAA/PVP sponges or films. Significance in difference between two groups was tested by Student *t*-test. (mean ± SD, * *p* < 0.05, ** *p* < 0.01).

When the porous PAA/PVP complex sponge came into contact with water, it rapidly swelled to form a bioadhesive hydrogel, indicating its ability to adhere to bio-tissues in a considerably short contact time. The shear adhesion strength of the PAA/PVP complex sponges with a synthetic leather piece after contact for 1–30 s was evaluated and compared with that of PAA/PVP complex films. A PAA/PVP complex sponge sheet was prepared with a swelling time of 60 min. The PAA/PVP complex film or sponge was kept in contact with the leather for 1–30 s without pressing, and the force required to detach the complex from leather was measured. The complex of the porous sponge form immediately swelled and adhered to the wetted leather piece with adhesion strength of 0.36 N/cm^2^ within 1 s. The force required for detachment gradually increased with increasing contacting time up to 0.9 N/cm^2^ at 20 s and decreased thereafter (Figure 2). In contrast, the film of the PAA/PVP complex showed lower adhesion strength than the sponge at a short contact time (10 s or less). It also increased with time and was higher than that of the sponge sheet after 30 s of contact.

**Figure 3:**
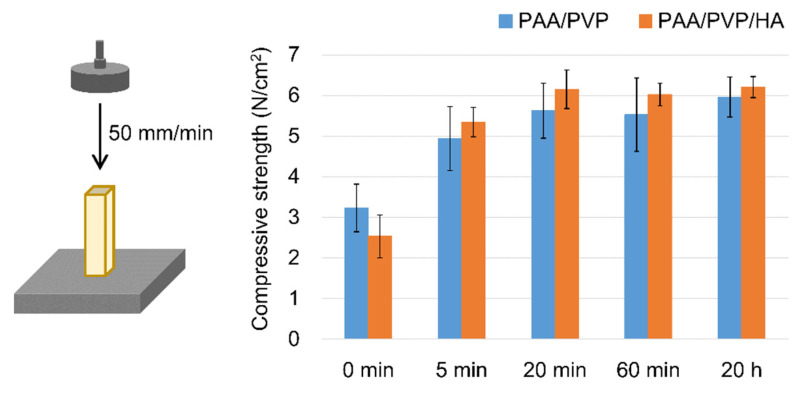
Compressive strength of PAA/PVP complexes prepared with or without HA freeze-dried after various swelling time. Data are presented as mean (SD).

As reported in our previous papers, water-swellable PAA/PVP complex films demonstrated preferable hemostatic effects in mice and rats [32,33]. The PAA/PVP complex sponges also exhibited adequate effect on hemostasis in mice (data not shown). However, considering its application as a hemostatic material in medical use, sufficient strength of the complex in both dried and swollen states is necessary. The enhanced mechanical strength of the dried sponge is desirable for holding using tweezers or pressing to the bleeding site. Adequate strength to maintain the shape of the swollen gel is required to make the gel stay in the hemorrhage area. HA was mixed with the complex to enhance its strength. HA is a water-soluble natural polysaccharide with a relatively rigid main chain structure. PAA/PVP/HA stick-form sponges were prepared by mixing HA with an aqueous PVP solution prior to addition to the PAA film. Sponges with or without HA were prepared with various swelling times, and their mechanical strength was measured using a compression test. The compressive strength of both sponge sticks increased with increasing swelling time and saturated at 20 min of swelling, regardless of whether HA was used. The addition of HA enhanced the strength of the dried sponge in the mean values; however, the difference was not significant (Figure 3).

**Figure 4:**
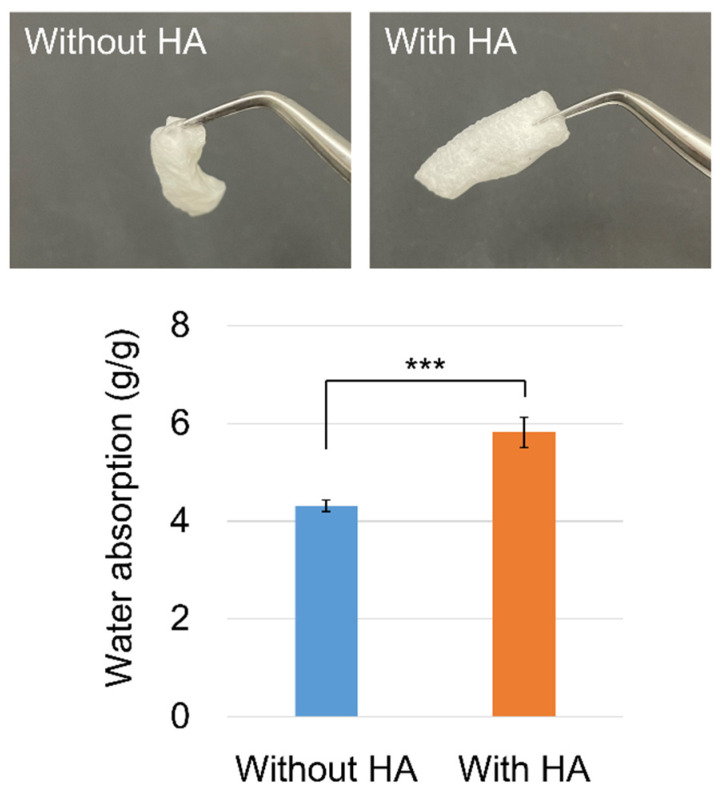
Swelling degree of PAA/PVP complexes with or without HA after being soaked in water for 10 s. Data were expressed as the weight ratio of absorbed water to the total weight of the polymers. Significance in difference between two groups was tested using Student’s *t*-test. (mean ± SD, *** *p* < 0.001).

**Figure 5:**
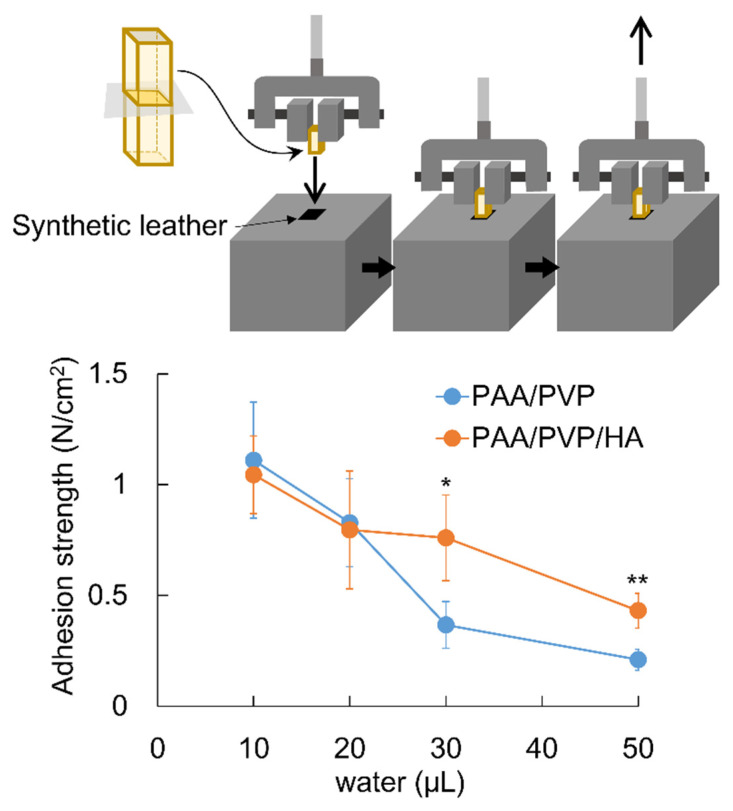
Pull-off adhesion strength of PAA/PVP complexes with or without HA with a synthetic leather piece wetted by various amount of water. Significance in difference between two groups was tested using Student’s *t*-test. (mean ± SD, * *p* < 0.05, ** *p* < 0.01).

A remarkable improvement in the strength was observed in the swollen state. After soaking in water for 10 s, the PAA/PVP sponges without HA almost lost their shape, whereas the PAA/PVP/HA sponges maintained their shape, although they swelled to a higher degree (Figure 4). The strength of the swollen gel affects the adhesion strength because gel collapse causes separation with low breaking strength. The pull-off adhesion strength with the wetted synthetic leather was then evaluated for the PAA/PVP complexes with or without HA. Complexes in stick form were prepared, and the cut surface was attached to the synthetic leather, which was wetted by a given amount of water. The force required to detach was significantly dependent on the amount of water, and both complexes showed a higher adhesion strength with a smaller amount of water, irrespective of the presence of HA (Figure 5). When the synthetic leather was wetted with a small amount of water (10 or 20 μL), both complex sticks exhibited similar adhesion strengths. A significant difference was observed when more than 30 μL water was used. PAA/PVP complexes containing HA exhibited twice as high adhesivity as those containing only PAA and PVP. HA does not contribute significantly to the adhesion force itself. The higher detaching forces were caused by the enhancement of the mechanical strength of the complex sponge in the swollen state.

In our previous study [35], we prepared the water-swellable PAA/PVP complex via another method where highly diluted PAA- and PVP-solutions were mixed under the presence of HA. After freeze-drying of the mixtures of PAA, PVP, and HA, smooth and flexible sponges were obtained, while those prepared without HA were weak and brittle. HA hindered the alignment of the polymer main chains and prevented the aggregation of PAA/PVP complexes. The improved strength of the HA-containing gels shown in Figure 4 was contributed by a rigid structure of HA molecule, or a suppression of the induction of heterogeneity by aggregation.

**Figure 6:**
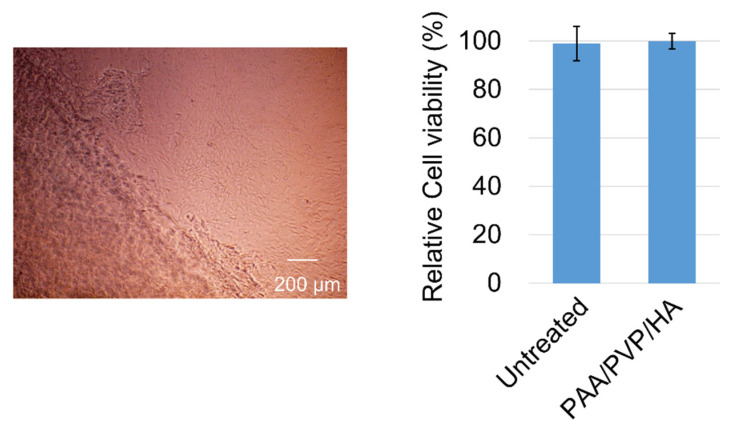
Viability and microscopic image of the fibroblasts cultured with a solid PAA/PVP/HA sponges. Data are presented as mean (SD).

Cytotoxicity of the PAA/PVP/HA complex sponge was examined in cultured mouse fibroblasts. The solid sponges were placed on the cells, and the medium was soon added to the wells. The sponges were immediately swollen in the wells, and then degraded slowly. On the next day, in the microscopic images, the living cells were observed even at the edges of the sponges. After incubating the cells with the swollen hydrogel for 3 days, WST-1 assay was performed. Compared with the control (untreated) cells, almost the same number of the cells were still alive (Figure 6). Under pH-neutral conditions, the PAA/PVP/HA complex is gradually degraded into water-soluble polymers which have been accepted as non-bioreactive with low toxicity, and safely used in pharmaceutical preparations. It attributes to the high biocompatibility of the PAA/PVP/HA complex hydrogel.

Bio-degradable PAA/PVP/HA complex consisting of only polymers with high biosafety has a potential for use in medical devices that are used by being implanted inside the body. In some applications, such as hemostatic materials used in surgeries and as an antiadhesive material to prevent postoperative wound adhesions, adhesive properties to a biotissue is often required. PAA/PVP/HA hydrogels which can adhere to mucous and serous membranes have the performance to meet those requirements, and their development will be the future research target of the material.

Prior to the clinical studies, the hemostatic effect of the cotton-backed PAA/PVP/HA sponge sheet was examined in the incised femoral veins of the mice. The PAA/PVP/HA sponge sheet adhered not only to biotissues, but also to the wetted metal instruments and wet rubber gloves. PAA/PVP/HA sheets (25 mm × 25 mm) were backed with a cotton lining for ease of handling. To evaluate the hemostatic effect in patients taking antithrombotic drugs, mice were injected with 50 IU of Fragmin, a low-molecular-weight heparin. The femoral vein was exposed under anesthesia and then cut. The cotton-backed PAA/PVP/HA sponge sheet placed on the bleeding site exhibited an outstanding hemostatic effect similar to that of PAA/PVP complex films presented in a previous paper [33]. The PAA/PVP/HA sponge sheet immediately absorbed the blood to an adhesive hydrogel, stuck tightly to the hemorrhage site, and arrested bleeding within minutes. The hemostatic efficacy of stick-type PAA/PVP/HA sponges was also examined. The stick strongly adhered to the bleeding site, and hemostasis was achieved soon after.

**Figure 7:**
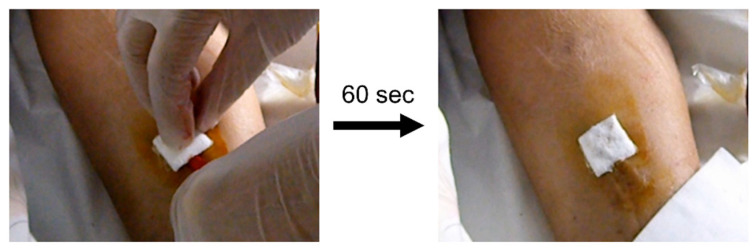
Hemostasis after blood dialysis with a PAA/PVP/HA sponge sheet with cotton lining.

A clinical study on hemostasis after blood dialysis was performed using a PAA/PVP/HA sponge sheet with a cotton lining. After dialysis, the puncture site was wiped with iodine solution, and a sponge sheet was placed on the wetted skin surface around the injection spot. After the needle was removed, the sponge sheet was gently pressed for 60 s. During treatment, blood oozing through the sponge sheet was not observed. The sponge was then gently peeled off and complete hemostasis was confirmed (Figure 7).

A cotton-backed PAA/PVP/HA sponge sheet is desirable for the treatment of skin-surface or fingertip injuries. The sponge can adhere to a surface wound wetted with water or an antiseptic solution. The soft sponge sheet could easily cover and stick to the fingertip without requiring a bandage or adhesive plaster. Many patients whose wounds were treated with the sponge sheet confirmed that the sponge greatly reduced pain. Dressing of the wound with a soft hydrogel can reduce pain by protecting the nerve endings from exposure to air [36]. The PAA/PVP/HA sponge sheet swollen by body fluid is a favorable wound dressing to protect the wound and reduce pain.

**Figure 8:**
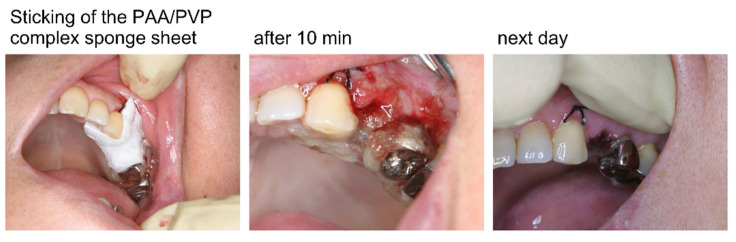
Hemostasis after tooth extraction with a PAA/PVP/HA sponge sheet in the patient taking warfarin (INR 1.69).

A PAA/PVP sponge sheet was used for hemostasis after tooth extraction. Soon after tooth extraction, a sponge sheet was attached to the socket. The sponge absorbed blood and saliva and immediately formed a gel. The swollen gel firmly adhered to the bleeding site to arrest hemorrhage effectively in all 11 patients, two of whom were taking antithrombotic drugs (Figure 8). Post-bleeding was not observed in any of the patients. The PAA/PVP complex sponge was stuck to the socket itself and did not require stitches, which would adversely affect local blood circulation, causing pain and delayed healing. Removal of the swollen complex hydrogel was not required because it slowly dissolved in the pH-neutral saliva and disappeared within days.

Hemostasis after tooth extraction was also attempted with the PAA/PVP/HA sponge in stick form (7 mm × 7 mm × 25 mm). The complex stick inserted into the socket soon swelled, and the hydrogel adhered to the bleeding tissue to effectively stop the hemorrhage. Although these results described in this report on the studies in a small number of patients do not demonstrate statistically significant efficacy, they exhibited the high potential of the PAA/PVP complex sponges as novel hemostatic materials. Rapid and clean healing was observed in the patients treated with the PAA/PVP complex. The complex hydrogel swelled by absorbing the blood contained platelets. The slow release of epidermal growth factor from the complex was confirmed in another study (data not shown). An investigation of the wound-healing effect and its detailed mechanism is currently underway.

## 4. Conclusions

A novel bioadhesive hydrogel consisting of PAA and PVP was prepared using the solid/solution interface complexation method. Freeze-drying of the hydrogel rendered a soft water-swellable sponge, and the potential for its application as a hemostatic agent was examined. The PAA/PVP sponge could be re-swelled by water to a bioadhesive gel and quickly adhered to tissues. The addition of HA improved the mechanical characteristics of the complex sponges. PAA/PVP/HA sponges showed high hemostatic efficiency in clinical studies after blood dialysis or tooth extraction, even in patients taking antithrombotic drugs. The bioadhesive PAA/PVP/HA sponge consists of low-toxicity polymers and has significant potential for future use.

## Data Availability

Not applicable.

## Supplementary Materials

The following supporting information can be downloaded at https://www.mdpi.com/article/10.3390/bioengineering9120755/s1, Figure S1: Influence of water amount on the pull-off adhesion strength of the PAA/PVP complex film. The PAA/PVP complex film was placed on a slightly wetted synthetic leather piece (10 mm × 10 mm) and pressed at a force of 5 N for 10 s. The upper support was then raised at 15 mm/min, and the force applied to detach or break the complex was recorded.
