# Supplementary material for: Water-Absorbing Bioadhesive Poly(Acrylic Acid)/Polyvinylpyrrolidone Complex Sponge for Hemostatic Agents
Source: Bioengineering (Basel). 2022 Dec 2;9(12):755. doi: 10.3390/bioengineering9120755 (PMC9774169; doi:10.3390/bioengineering9120755)
Supplement: Supplementary file 1 [file bioengineering-09-00755-s001.zip › bioengineering-2028163-SI.pdf]

Supplementary Materials

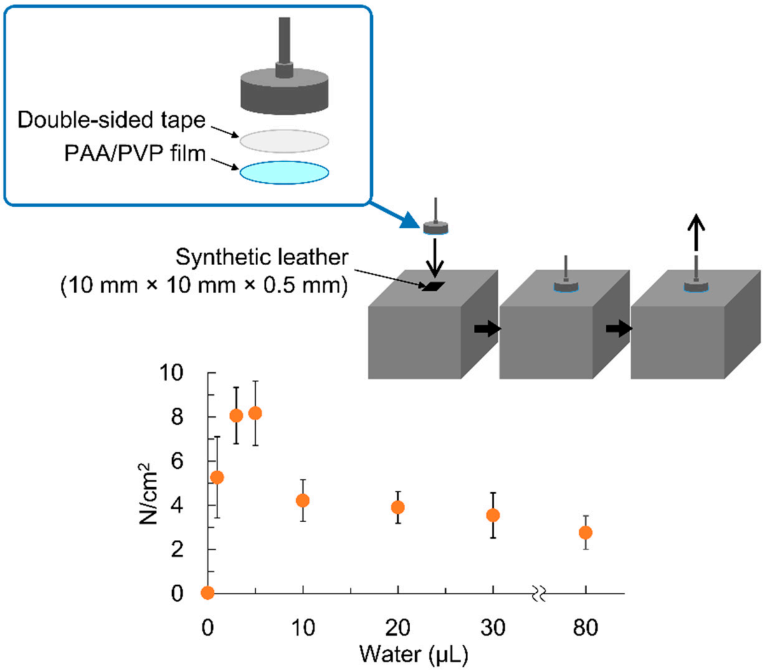

Figure S1: Influence of water amount on the pull-off adhesion strength of the PAA/PVP complex film. The PAA/PVP complex film was placed on a slightly wetted synthetic leather piece (10 mm × 10 mm) and pressed at a force of 5 N for 10 s. The upper support was then raised at 15 mm/min, and the force applied to detach or break the complex was recorded.
